# Acute cholangitis due to haemobilia complicating percutaneous cholecystostomy: First literature case report

**DOI:** 10.1016/j.ijscr.2022.107273

**Published:** 2022-06-08

**Authors:** Hazem Beji, Souhaib Atri, Houcine Maghrebi, Anis Haddad, Amin Makni, Montasser Kacem

**Affiliations:** Department of General Surgery A, Hospital La Rabta, Tunis, Tunisia; University Tunis El Manar, Faculty of Medicine of Tunis, Tunisia

**Keywords:** Percutaneous cholecystostomy, Haemobilia, Acute cholangitis, Case report

## Abstract

**Background:**

Laparoscopic cholecystectomy is the standard treatment for acute cholecystitis. Cholecystostomy is a good option in patients with significant comorbidities. We report a case of a patient having had a percutaneous cholecystostomy for acute cholecystitis complicated with haemobilia and acute cholangitis.

**Presentation of a case:**

A woman aged 64 years old, with a history of diabetes, arterial hypertension, and chronic obstructive pulmonary disease was admitted to our institution with acute cholecystitis.

We opted for transhepatic percutaneous cholecystostomy (PC) and antibiotics.

On the fourth day, the patient had acute cholangitis due to haemobilia.

We injected physiologic saline serum through the drain of cholecystostomy to dissolve the blood clot. There was a clinical improvement.

We performed laparoscopic cholecystectomy two months later. The patient had an uneventful recovery with a follow-up of five months.

**Discussion:**

We report the first literature report of acute cholangitis due to haemobilia complicating percutaneous cholecystostomy in a patient admitted for cholecystitis.

We highlight the importance of the injection of saline physiologic serum from the catheter. Medical treatment with antibiotics may be enough knowing that blood clots can disappear spontaneously. In case of failure, ERCP with sphincterotomy should be performed.

**Conclusion:**

Haemobilia causing acute cholangitis is a rare complication of percutaneous cholecystostomy. Conservative treatment with antibiotics and injection of saline physiologic serum from the catheter is a good treatment option. In case of failure, ERCP should not be delayed.

## Highlights

• Cholecystostomy for acute cholecystitis is a considerable option in comorbid patients.
• Acute cholangitis due to haemobilia is an extremely rare complication.
• Injection of physiologic serum saline from the drain is a conservative and efficient treatment.
• It facilitates the dissolution of blood clots.

## 1 Background

Laparoscopic cholecystectomy is the standard treatment for acute cholecystitis [1]. In patients with significant comorbidities and high operative risk, percutaneous cholecystostomy (PC) can be a good option to avoid perioperative morbidity and mortality. It is considered a safe procedure with complication rates of 0–16 % [2]. Haemobilia causing acute cholangitis is an extremely rare complication that was never described in the literature. We report a case of a patient having had a percutaneous cholecystostomy for acute cholecystitis complicated with haemobilia and acute cholangitis.

This work has been reported in line with the SCARE 2020 criteria [3].

## 2 Presentation of a case

A woman aged 64 years old, with a history of diabetes, arterial hypertension, and chronic obstructive pulmonary disease was admitted to our institution due to acute constant pain localized in the right upper abdomen evolving for three days.

On the physical examination at admission, she had a fever of 39 degrees. Her blood pressure was 100/60 mmHg and pulse rate 110/min, regular. Abdominal examination revealed tenderness in the right upper quadrant with a positive Murphy's sign.

Laboratory studies revealed leukocytosis with 19,500/mm^3^ and elevation of C reactive protein to 22 mg/L. Prothrombin time, bilirubin level, and liver enzymes were normal.

Abdominal ultrasound showed the presence of multiple gallstones with a wall thickening and pericholecystic abscess of 3 cm.

The diagnosis of acute cholecystitis was confirmed. Due to the age and comorbidities of the patient, we opted for transhepatic percutaneous cholecystostomy (PC) and antibiotics.

Initially, there was a clinical improvement. But on the fourth day, the patient presented abdominal pain, fever, and jaundice.

**Fig. 1:**
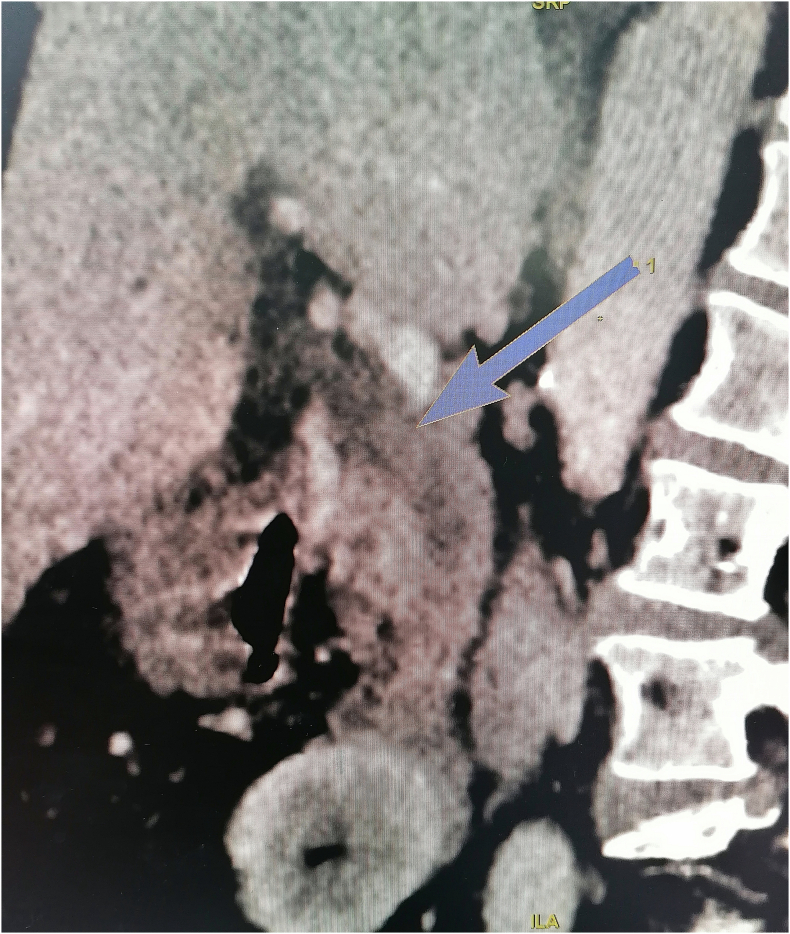
Sagittal CT scan view showing high-density material in the common bile duct.

Laboratory findings showed leukocytosis with 21,200/mm^3^ and elevation of C reactive protein to 21 mg/L, Total bilirubin/direct bilurbin = 86 μmol/L/78 μmol/L, AST/ALT = 128/95. We made a computed tomography (CT) scan showing blood within the common bile duct appearing as high-density material (Fig. 1).

We injected 5 cubic centimeters (cc) of physiologic saline serum through the drain of cholecystostomy three times per day to dissolve the blood clot. There was a clinical improvement with the disappearance of fever and jaundice after five days.

In laboratory findings, there was normalization of liver enzymes and total bilirubin.

**Fig. 2:**
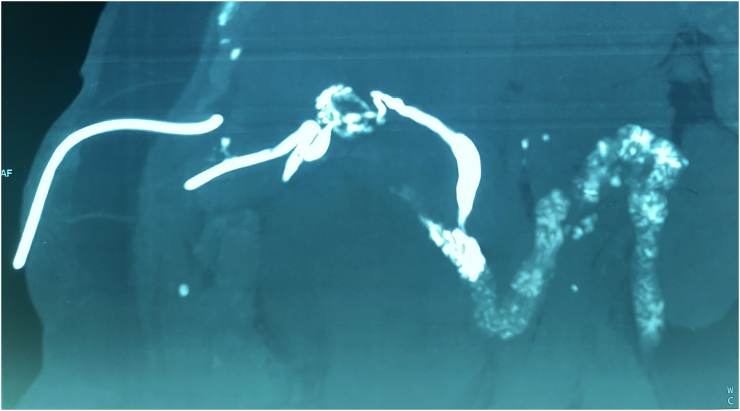
MRCP shows vacuity of the common bile duct.

We made a magnetic resonance cholangiopancreatography (MRCP) which confirmed the vacuity of the bile common duct (Fig. 2). The drain of cholecystostomy was removed after one month.

We performed laparoscopic cholecystectomy two months later.

The patient had an uneventful recovery with a follow-up of five months.

## 3 Discussion

We reported the first literature report of acute cholangitis due to hemobilia complicating percutaneous cholecystostomy in a patient admitted for calculous cholecystitis. We successfully treated this complication by injections of physiologic serum saline from the drain to make the dissolution of the blood clot.

One of the weaknesses of our work is the inaccessibility of the emergency MRI exam.

Laparoscopic cholecystectomy is the gold standard treatment for cholecystitis. PC has emerged in the last decades as an alternative treatment to decrease morbidity and mortality related to surgery in elderly and comorbid patients [4], [5], [6], [7]. This technique allows bile drainage for patients initially unfit for surgery. Once the patient's condition has improved, laparoscopic cholecystectomy is performed within two months [8], [9].

Complications associated with percutaneous cholecystostomy include catheter dislocations, bile leakage, hemorrhage, and perforation of the intestinal loop [10], [11], [12].

Acute cholangitis secondary to haemobilia is an extremely rare complication that was never reported in the literature. The main symptoms are recurrence of abdominal pain with fever and jaundice.

MRCP is the best exam to confirm hemobilia [13], [14]. In our case, haemobilia was due to a hemorrhage in the gallbladder wall. A blood clot migrated from the gallbladder to the common bile duct and caused acute cholangitis.

Endoscopic retrograde cholangiopancreatography (ERCP) is considered to be an excellent diagnostic and treatment modality for hemobilia. In case of failure, percutaneous transhepatic biliary drainage can be performed. Blood clots generally disappear afterward [15]. Endoscopic nasobiliary drainage (ENBD) also presents a therapeutic alternative [16].

In our case, we opted to inject carefully physiologic serum saline from the drain to make the dissolution of the blood clot faster. Our procedure presents a risk of migration of gallstones into the common bile duct which would cause recurrence of acute cholangitis. We tried to minimize that risk by injecting 15 mL of physiologic serum saline and splitting it into three injections per day.

In summary, acute cholangitis secondary to haemobilia, complicating percutaneous cholecystostomy is a very rare situation. We highlight the importance of the injection of saline physiologic serum from the catheter. Otherwise, medical treatment with antibiotics may be enough knowing that blood clots can disappear spontaneously. In the absence of clinical improvement, ERCP with sphincterotomy should be performed.

## 4 Conclusion

Percutaneous cholecystostomy is a safe procedure for acute cholecystitis in patients with comorbidities. Haemobilia causing acute cholangitis is an extremely rare complication. Conservative treatment with antibiotics and injection of saline physiologic serum from the catheter is a good treatment option. In case of failure, ERCP should not be delayed.

## Sources of funding

This research did not receive any specific grant from funding agencies in the public, commercial, or not-for-profit sectors.

## Ethical approval

Not required.

## Consent

Written informed consent was obtained from the patient for publication of this case report and accompanying images. A copy of the written consent is available for review by the Editor-in-Chief of this journal on request.

## Provenance and peer review

Not commissioned, externally peer reviewed.

## Author contribution

Hazem Beji and Souhaib Atri did the conception and design of the work, the data collection, and the data analysis and interpretation.

Houcine Maghrebi and Anis Haddad did the critical revision of the article

Amin Makni and Montassar Kacem did the final approval of the version to be published.

## Registration of research studies

Not applicable.

## Guarantor

Dr. Beji Hazem.

Dr. Souhaib Atri.

## Declaration of competing interest

No conflicts of interest.

## References

[1] Okamoto K., Suzuki K., Takada T. (2018 Jan). Tokyo Guidelines 2018: flowchart for the management of acute cholecystitis. J. Hepatobiliary Pancreat. Sci..

[2] Turiño S.Y., Shabanzadeh D.M., Eichen N.M., Jørgensen S.L., Sørensen L.T., Jørgensen L.N. (2019 Feb). Percutaneous cholecystostomy versus conservative treatment for acute cholecystitis: a cohort study. J. Gastrointest. Surg..

[3] Agha R.A., Franchi T., Sohrabi C., Mathew G., for the SCARE Group (2020). The SCARE 2020 guideline: updating consensus Surgical CAse REport (SCARE) guidelines. Int. J. Surg..

[4] Stanek A., Dohan A., Barkun J., Barkun A., Reinhold C., Valenti D., Cassinotto C., Gallix B. (2018 Sep). Percutaneous cholecystostomy: a simple bridge to surgery or an alternative option for the management of acute cholecystitis?. Am. J. Surg..

[5] Casola G. (1992). Percutaneous gallbladder puncture and cholecystostomy: results, complications and caveats for safety. Radiology.

[6] Gervais D.A., Mueller P.R. (1996). Percutaneous cholecystostomy. Semin. Intervent. Radiol..

[7] Van Overhagen H., Meyers H., Tilanus H.W., Jeekel J., Lameris J.S. (1996). Percutaneous cholecystostomy for patients with acute cholecystitis and an increased surgical risk. Cardiovasc. Intervent. Radiol..

[8] Itoi T., Tsuyuguchi T., Takada T. (2013). TG13 indications and techniques for biliary drainage in acute cholangitis (with videos). J. Hepatobiliary Pancreat. Sci..

[9] Yamashita Y., Takada T., Kawarada Y. (2007). Surgical treatment of patients with acute cholecystitis: Tokyo guidelines. J. Hepato-Biliary-Pancreat. Surg..

[10] Akhan O., Akinci D., Ozmen M.N. (2002 Sep). Percutaneous cholecystostomy. Eur. J. Radiol..

[11] Colonna A.L., Griffiths T.M., Robison D.C., Enniss T.M., Young J.B., McCrum M.L., Nunez J.M., Nirula R., Hardman R.L. (2019 Jun). Cholecystostomy: are we using it correctly?. Am. J. Surg..

[12] Gervais D.A., Mueller P.R. (1996). Percutaneous cholecystostomy. Semin. Intervent. Radiol..

[13] Asselah T., Condat B., Sibert A. (2001). Haemobilia causing acute pancreatitis after percutaneous liver biopsy: diagnosis by magnetic resonance cholangiopancreatography. Eur. J. Gastroenterol. Hepatol..

[14] Cathcart S., Birk J.W., Tadros M., Schuster M. (2017 Oct). Hemobilia: an uncommon but notable cause of upper gastrointestinal bleeding. J. Clin. Gastroenterol..

[15] Kim K.H., Kim T.N. (2012 Apr). Etiology, clinical features, and endoscopic management of hemobilia: a retrospective analysis of 37 cases. Korean J. Gastroenterol..

[16] Arata R., Yanagawa S., Miyata Y., Ishitobi T., Kodama S., Sumimoto K. (2020). Hemobilia after laparoscopic cholecystectomy that was successfully treated conservatively: case report. Int. J. Surg. Case Rep..

